# Deep learning identifies Acute Promyelocytic Leukemia in bone marrow smears

**DOI:** 10.1186/s12885-022-09307-8

**Published:** 2022-02-22

**Authors:** Jan-Niklas Eckardt, Tim Schmittmann, Sebastian Riechert, Michael Kramer, Anas Shekh Sulaiman, Katja Sockel, Frank Kroschinsky, Johannes Schetelig, Lisa Wagenführ, Ulrich Schuler, Uwe Platzbecker, Christian Thiede, Friedrich Stölzel, Christoph Röllig, Martin Bornhäuser, Karsten Wendt, Jan Moritz Middeke

**Affiliations:** 1 Department of Internal Medicine I, University Hospital Carl Gustav Carus, 01307 Dresden, Saxony, Germany; 2 Institute of Software and Multimedia Technology, Technical University Dresden, Dresden, Germany; 3 Department of Medicine I, Hematology, Cellular Therapy, Hemostaseology, University of Leipzig, Leipzig, Germany; 4 German Consortium for Translational Cancer Research, Heidelberg, Germany; 5 National Center for Tumor Disease (NCT), Dresden, Germany

**Keywords:** Acute promyelocytic leukemia, Acute myeloid leukemia, Deep learning, Artificial intelligence, Bone marrow smear

## Abstract

**Background:**

Acute promyelocytic leukemia (APL) is considered a hematologic emergency due to high risk of bleeding and fatal hemorrhages being a major cause of death. Despite lower death rates reported from clinical trials, patient registry data suggest an early death rate of 20%, especially for elderly and frail patients. Therefore, reliable diagnosis is required as treatment with differentiation-inducing agents leads to cure in the majority of patients. However, diagnosis commonly relies on cytomorphology and genetic confirmation of the pathognomonic t(15;17). Yet, the latter is more time consuming and in some regions unavailable.

**Methods:**

In recent years, deep learning (DL) has been evaluated for medical image recognition showing outstanding capabilities in analyzing large amounts of image data and provides reliable classification results. We developed a multi-stage DL platform that automatically reads images of bone marrow smears, accurately segments cells, and subsequently predicts APL using image data only. We retrospectively identified 51 APL patients from previous multicenter trials and compared them to 1048 non-APL acute myeloid leukemia (AML) patients and 236 healthy bone marrow donor samples, respectively.

**Results:**

Our DL platform segments bone marrow cells with a mean average precision and a mean average recall of both 0.97. Further, it achieves high accuracy in detecting APL by distinguishing between APL and non-APL AML as well as APL and healthy donors with an area under the receiver operating characteristic of 0.8575 and 0.9585, respectively, using visual image data only.

**Conclusions:**

Our study underlines not only the feasibility of DL to detect distinct morphologies that accompany a cytogenetic aberration like t(15;17) in APL, but also shows the capability of DL to abstract information from a small medical data set, i. e. 51 APL patients, and infer correct predictions. This demonstrates the suitability of DL to assist in the diagnosis of rare cancer entities. As our DL platform predicts APL from bone marrow smear images alone, this may be used to diagnose APL in regions were molecular or cytogenetic subtyping is not routinely available and raise attention to suspected cases of APL for expert evaluation.

**Supplementary Information:**

The online version contains supplementary material available at 10.1186/s12885-022-09307-8.

## Background

Acute promyelocytic leukemia (APL) is a distinct subclass of acute myeloid leukemia (AML) which is characterized by a reciprocal and balanced translocation between the promyelocytic leukemia protein (PML) gene on chromosome 15 and the retinoic acid receptor α (RARα) gene on chromosome 17 [1, 2]. The t(15;17) results in an oncogenic fusion protein PML-RARα which functions as a transcriptional repressor of RARα target genes and impairs the homeostatic function of PML thereby promoting a proliferation of myeloid progenitor cells and provoking a maturation arrest at the promyelocytic stage [3–5]. APL was first described by the Norwegian hematologist Leif Hillestad in 1957 [6] and for a long time it was considered one of the most lethal leukemias [7] with population-based incidence rates varying between different ethnicities [8–10]. The introduction of all-*trans* retinoic acid (ATRA) [11] and arsenic trioxide (ATO) [12] has revolutionized APL therapy and outcome nowadays showing remarkable cure rates [13, 14]. Nevertheless, APL is considered a hematologic emergency and requires immediate treatment upon suspected diagnosis, both causally and supportive, due to possible early death from bleeding [13]. Early death rates in APL – commonly defined as death within 30 days of presentation [15] – appear to be underestimated in the medical literature: While clinical trials frequently show early death rates below 10% it has to be considered that a substantial number of patients even dies before APL is diagnosed and patients with significant comorbidities or higher age are often excluded from trials leading to bias [15, 16]. In patients ineligible for clinical trials, registry data as well as population-based analyses show an early death rate of approximately 20% with even higher rates for elderly patients [15–19]. When diagnosed and treated promptly, APL is curable in the majority of patients. Therefore, fast and accurate diagnosis as well as immediate treatment upon suspicion is crucial [13]. Classical APL can be recognized by a distinct morphology of abnormal promyelocytes with a heavy granulation pattern and characteristic cells containing single Auer rods or bundles of Auer rods in the cytoplasm (‘faggot cells’) [20]. Therefore, cytomorphologic assessment by experienced hemtopathologists is essential for APL diagnosis since it is fast, feasible and can often reinforce clinically suspected diagnosis. Still, diagnosis of APL routinely encompasses cytomorphology [21, 22] as well as cytogenetics for confirmation of suspected diagnosis [13], however genetic analyses take more time and resources until results are available. Further, high-quality genetic testing might not be ubiquitously available.

Machine Learning (ML), especially Artificial Neural Nets (ANN), can handle large-scale data sets and are implemented as image recognition and computer vision technologies, especially Convolutional Neural Nets (CNN) [23, 24] as a form of Deep Learning (DL). DL models consist of massive parallel computing systems consisting of large numbers of interconnected processing units called artificial neurons, [25, 26] which can be run efficiently on high performance computing systems. CNNs contain multiple neural layers to provide functionality for image recognition [24] Thus, these capabilities can be utilized for cell segmentation, cell recognition and disease classification in hematological malignancies [27–29]. We here present a CNN-based scalable approach that can detect APL among healthy bone marrow donor and non-APL AML samples from bone marrow smear (BMS) images. The resulting models provide a reliable method for APL diagnosis when genetic data are still pending or an experienced hematopathologist is not immediately available, thereby reducing treatment delay. Further, our DL model can be implemented remotely in areas where no immediate access to high-quality genetic testing is available, thereby enabling the diagnosis of APL in non-industrialized countries, where APL is often more common [8, 9].

## Methods

In this study, we trained a multi-stage DL platform to segment cells in BMS and distinguish between APL and non-APL AML as well as APL and healthy bone marrow donor samples using visual image data only.

### Data set and molecular analysis

We retrospectively identified 58 APL patients that have been diagnosed and treated in the multicentric AIDA2000 (NCT00180128) [30] and NAPOLEON studies (national APL observational study, NCT02192619) [31] or from the German Study Alliance Leukemia (SAL) registry (NCT03188874). Eligibility criteria for the APL cohort were newly diagnosed APL according to WHO criteria [32] (FAB M3) as defined by the presence of t(15;17) or fusion transcript *PML*-*RARA*, age ≥ 18 years and available biomaterial at diagnosis. Diagnosis of APL was confirmed using standard techniques for chromosome banding and fluorescence in situ hybridization (FISH). Seven samples were excluded because BMS were inconclusive due to dry tap and diagnosis was performed using peripheral blood. 51 APL BMS were analyzed for the purpose of this study. The first control cohort was comprised of 236 bone marrow samples from healthy bone marrow donors who underwent bone marrow donation at our center. The second control cohort consisted of 1048 BMS from patients with non-APL AML that were identified from the multicentric German SAL registry. Written informed consent was obtained from all patients and donors according to the Declaration of Helsinki. The studies were previously approved by the Institutional Review Board of the Technical University Dresden (EK 98032010). High-resolution pictures of representative areas of the BMS were taken using the Nikon ECLIPSE E600 microscope (50-fold magnification) with the Nikon DSFi2 mounted camera and Nikon Imaging Software Elements D4 for image processing. For each sample, one image of a representative area was taken for evaluation by the deep learning model. To account for imbalances in the data sets, image augmentation techniques were employed as described below.

### Deep learning model

#### Pre-processing and cell segmentation

**Fig. 1:**
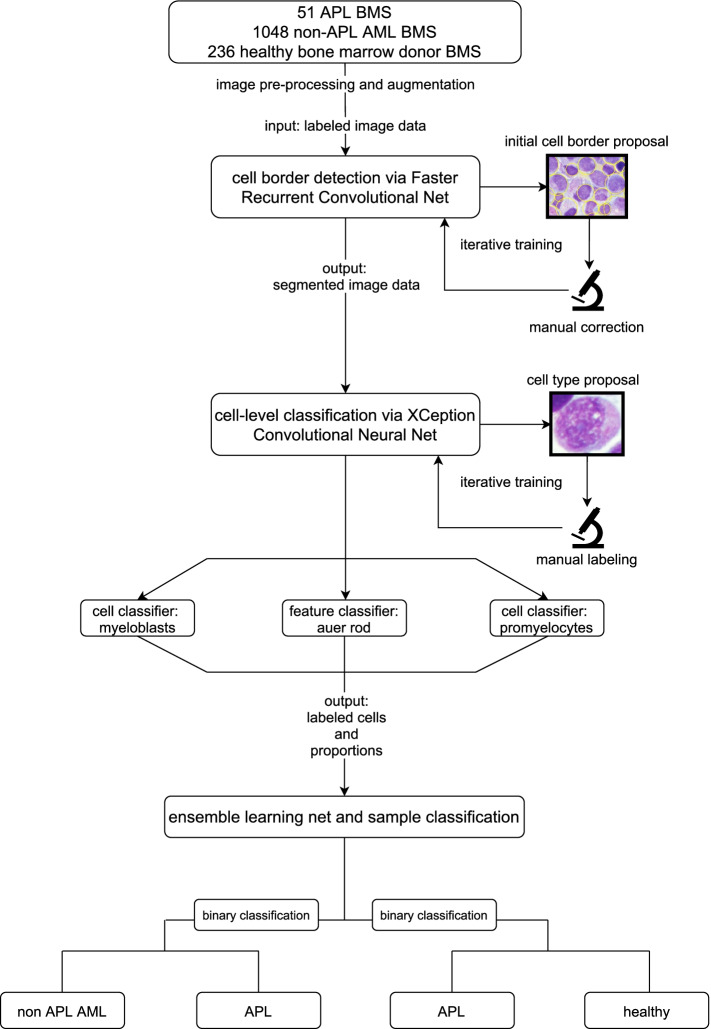
Workflow of the multi-step deep learning model for APL recognition. We identified patients with APL, non-APL AML and healthy bone marrow donors by retrospective chart review. Representative images of bone marrow smears (BMS) were labeled according to diagnosis. After image preprocessing, transformation and augmentation, initial cell border proposals were given by the Faster Region-based Convolutional Neural Net (FRCNN) that were manually corrected on an online segmentation and annotation platform based on the VGG image annotator tool. The FRCNN was trained iteratively to improve cell border proposals. Segmented cells were manually labeled according to cell type (myeloblasts, promyelocytes) and Auer rods. Convolutional neural nets were then implemented on the automatically segmented cells for binary classification of individual cell types and features. Their output was used to train an ensemble neural net for the binary classification between APL and non-APL AML or APL and healthy bone marrow donor samples

We developed a multi-step ML workflow with individual DL models for different tasks as shown in Fig. 1. After digitization, BMS images were uploaded to an online segmentation and labeling platform that we developed for the purpose of this work. The platforms architecture was designed to receive BMS images sized 2560 * 1920 pixels as input at the top level for BMS images while receiving 299*299 pixels input size on the level of individual cells in subsequent cell-level classification tasks (see below). Picture input at the top level corresponded to an area of 171*128 µm. In the first step, initial cell segmentation was performed with a human-in-the-loop approach by hematologists with the VGG Image Annotator [33] tool to train a Faster Region-based Convolutional Neural Net [34] (FRCNN). Cell borders were initially drawn by hematologists, then, the FRCNN learned by example and subsequently provided cell border proposals on unsegmented cells that were manually corrected in an iterative way. Thereby, the FRCNN substantially improved its accuracy over iterations enabling the final model to automatically segment BMS images without the need for human manual correction. The trained FRCNN was used to subsequently segment cells on all available BMS images. Hyperparameter optimization was performed automatically using the Optuna [35] framework with a predefined hyperparameter space.

#### Cell labeling and cell-level classification

In the second step and analogous to training the model by example to detect cell borders, feature extraction was initially performed manually by hematologists, i.e., labels of cell type, lineage and distinct characteristics like Auer rods were attributed to 8500 individual cells by hematologists (Tab. S1 shows the numbers of individual labels). Cell size, volume and contrast were automatically calculated by computer vision algorithms. Given the rarity of the disease and therefore limited number of cases included in our study, image augmentation techniques like linear transformations, color shift and brightness adjustment were applied to increase sample size and balance samples for binary classifications as imbalances may otherwise have introduced bias towards the predominant class. For image classification, model architecture was based on the Xception CNN [36]. In order to deal with the limited sample size, we used transfer learning and modified the fully connected exit flow layers for the 2048-dimensional output vectors of the core Xception architecture between one and three fully connected layers with dropout. The final architectures were found via hyperparameter optimization and differed between individual models. We used a top-down approach of three different inter-connected CNNs that zoomed in on individual cells for different binary classification purposes. The hierarchical modeling was designed as follows: Cell-level CNNs were employed to classify myeloblasts, promyelocytes and Auer rods. For example, the myeloblast-detection-CNN was trained to fit the labels “myeloblast” and “non-myeloblast” to each cell that had been segmented by the FRCNN previously (in the first step). Simultaneously, the same cells were evaluated by the promyelocyte-detection-CNN and Auer-rod-detection-CNN. For instance, a promyelocyte with one single Auer rod would receive the following encoding: myeloblast 0; promyelocyte 1; Auer rod 1. This way, ratios of myeloblasts, promyelocytes and cells bearing Auer rods per BMS became available.

#### Image-level classification via ensemble learning

Subsequent results of the cell-level classification CNNs were put out as ratios (e. g. ratio of myeloblasts per BMS, ratio of promyelocytes per BMS). The output of the aforementioned CNNs was then combined via a fully connected layer without dropout to form binary ensemble neural nets (ENNs) producing the final labels according to the individual diagnosis, i. e. APL, non-APL AML or healthy donor. The final models again underwent hyperparameter optimization and were trained on the augmented training set with optimized hyperparameters. Then, cross-validation was performed on patient data that were withheld from model building and training using a 2:1 split to prevent patients from crossing between training and test set. In previous iterations, we tried to train a single CNN end-to-end on the BMS images for binary classification purposes, however, we found results to be unsatisfactory likely due to the small sample size of APL (*n* = 51). Yet, the implementation of hierarchical CNNs to first classify cells as a proxy and then implement their output for ensemble learning was efficient in dealing with the small sample size and yielded improved performance in comparison to a single-CNN approach. To implement the model to achieve satisfactory results, image augmentation, balancing of data sets for binary decisions (e. g. APL vs. healthy controls) and hierarchical learning were crucial. In summary, an image is first segmented by the FRCNN to discriminate between viable cells and background/smudge, then classified by the CNNs on the cell-level and lastly (based on the proportions per BMS) classified by the ENNs on the image-level for the respective diagnosis.

### Performance evaluation

Classification performance was assessed by precision, recall and F1-score with threefold cross-validation. Precision (positive predictive value) is defined as the fraction of true positives among all positive predictions while recall (sensitivity) is defined as the fraction of all positive predictions among all relevant events. The true positive rate (TPR) corresponds to sensitivity/recall while the false positive rate (FPR) corresponds to 1-specificity. The F1-score is a combined metric of both precision and recall. As both precision and positive predictive value as well as recall and sensitivity can be used interchangeably depending on the contextual domain, we will from now on use the terms precision and recall for the remainder of this manuscript. Area under the curve (AUC) was calculated for the receiver operating characteristic (ROC) and precision-recall-curves. Calculations and visualizations were performed in Python version 3.7.9.

### Data and code availability

The data supporting the conclusions of this article is available under https://www.kaggle.com/dataset/a49eb5eb219384adf92856e43dcfc79b9cf1eaea5ec13bd57ef304d173ebe42c All models were built in Python version 3.7.9 with Keras version 2.3.0 and TensorFlow version 2.1.2. Computations were performed using a high-performance computing system. The code supporting the conclusions of this article is available under https://github.com/SebastianRiechert/autofrcnn and https://github.com/TimSchmittmann/Fast-and-Accurate-Diagnosis-of-APL-from-BMS

## Results

Median age for the non-APL AML and APL cohort were 57 (IQR: 49–67) and 50.5 (42.3–58) years, respectively. The majority of cases were de novo AML/APL. Median bone marrow blast counts were 63.5% (IQR: 41.5–80) and 63 (53.5–76) for non-APL AML and APL, respectively. Table 1 provides detailed information on patient characteristics.

**Table 1:** Patient characteristics

parameter	non-APL AML	APL	Bone marrow donors
N	1 048	58	236
Age, median (IQR)	57 (49–67)	50.5 (42.25–58)	31 (25–39)
**Sex, %**			
Male	45.5	45.7	70
Female	54.5	54.3	30
**AML type, %**			
de novo	77.5	90.5	/
sAML	13.6	0	/
tAML	8.9	9.5	/
**ELN2017 risk, %**			
Favorable	34.1	/	/
intermediate	44.7	/	/
adverse	21.2	/	/
WBC in GPt/l, median (IQR)	14 (2.7–45.2)	1.3 (0.7–6.4)	/
Hb in mmol/l, median (IQR)	5.8 (5.0–6.8)	6.3 (5.3–6.9)	/
Hb in g/dl, median (IQR)	9.3 (8.1–11.0)	10.1 (8.5–11.1)	/
Plt in GPt/l, median (IQR)	56 (31–103)	27 (18–57)	/
PB blasts, median (IQR)	27 (6–62)	14.75 (1.3–63)	/
BM blasts, median (IQR)	63.5 (41.5–80)	63 (53.5–76)	/

Patient characteristics of non-APL AML, APL and control (bone marrow donors) groups. AML type was defined according to the WHO 2016 classification

*sAML* Secondary AML, *tAML* Therapy-associated AML, *WBC* white blood cell count, *Hb* Hemoglobin, *Plt* Platelet count, *PB* Peripheral blood, *BM* Bone marrow, *N* Number, *IQR* interquartile range

**Fig. 2:**
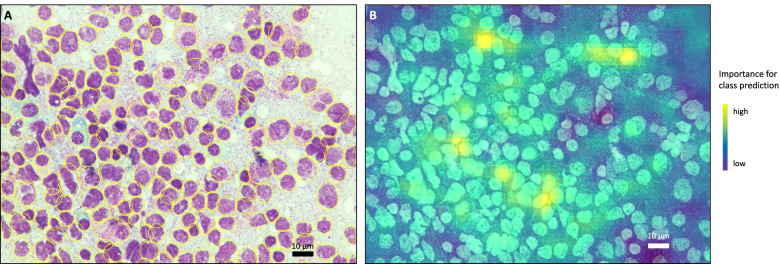
Examples of automated segmentation and occlusion sensitivity mapping. A Faster Recurrent Neural Network (FRCNN) was used for cell segmentation. First, it was trained by human example and after iterative learning, automated cell detection was performed (**A**). Segmented cells show a yellow elliptic border. With respect to explainable artificial intelligence, we used occlusion sensitivity mapping to retrace the decision-making process of the convolutional neural nets in image-level recognition (**B**). In occlusion sensitivity mapping, parts of the image are iteratively blocked from evaluation by the neural network and performance is measured. If the blocked part of the image is highly important for correct classifications, performance will drop accordingly. This process is iteratively repeated for the entire image. The result can be visualized in the sense that highly important image areas are highlighted (yellow/green) while less important or negligible areas are shaded (blue/purple)

94.162 individual cells were manually segmented to train the FRCNN. Segmentation achieved a mean average precision (mAP) and a mean average recall (mAR) of both 0.97 at an intersection-over-union-ratio of 0.5 and a mAP of 0.88 and mAR of 0.90 at an intersection-over-union-ratio of 0.5 to 0.95 (for increasingly strict overlaps between predicted cell boundaries and the ground truth). An example of automatically segmented bone marrow cells in an APL BMS can be seen in Fig. 2A. Rarely, inaccuracies were observable due to overlapping cells. Subsequently, automatically segmented cells were used for feature selection and classification. Using image augmentation, we obtained balanced data sets of 969 APL images and 944 healthy controls for the binary classification between APL and healthy bone marrow donor samples and 2550 APL images and 2500 non-APL AML images for the binary classification between APL and non-APL AML. To prevent overfitting, we used pooling dropout of 0.32 and 0.46, respectively, as suggested by automated hyperparameter optimization. The median number of cells per image was 169. Regarding time from image upload to final output, i. e. suspected diagnosis, it took the high-performance computing system 5 s to load an individual image. The subsequent segmentation via the FRCNN took on average 4.4 s and the consecutive classification of each individual cell via the CNNs took on average 0.2 s per cell. The integration of the CNNs results, i. e. the proportions of myeloblasts, promyelocytes and Auer rods per image via the ENN took 2.1 s on average. Given a median of 169 cells per image, the entire process from uploading the image to final output, i. e. suspected diagnosis per image, took 45.3 s on average. We used three different CNNs that were trained to detect cell types (myeloblasts, promyelocytes) and Auer rods based on individual binary classifications on the cell-level. Subsequently, this information was integrated by our ENN for sample classification. In terms of explainable AI, we created occlusion sensitivity maps of BMS image-level classification tasks to retrace the CNNs’ image evaluation. On the BMS image-level, we found the neural nets to be cell-specific in a proof-of-concept fashion, i. e. evaluating cells rather than background, smudge, or noise (Fig. 2B).

**Fig. 3:**
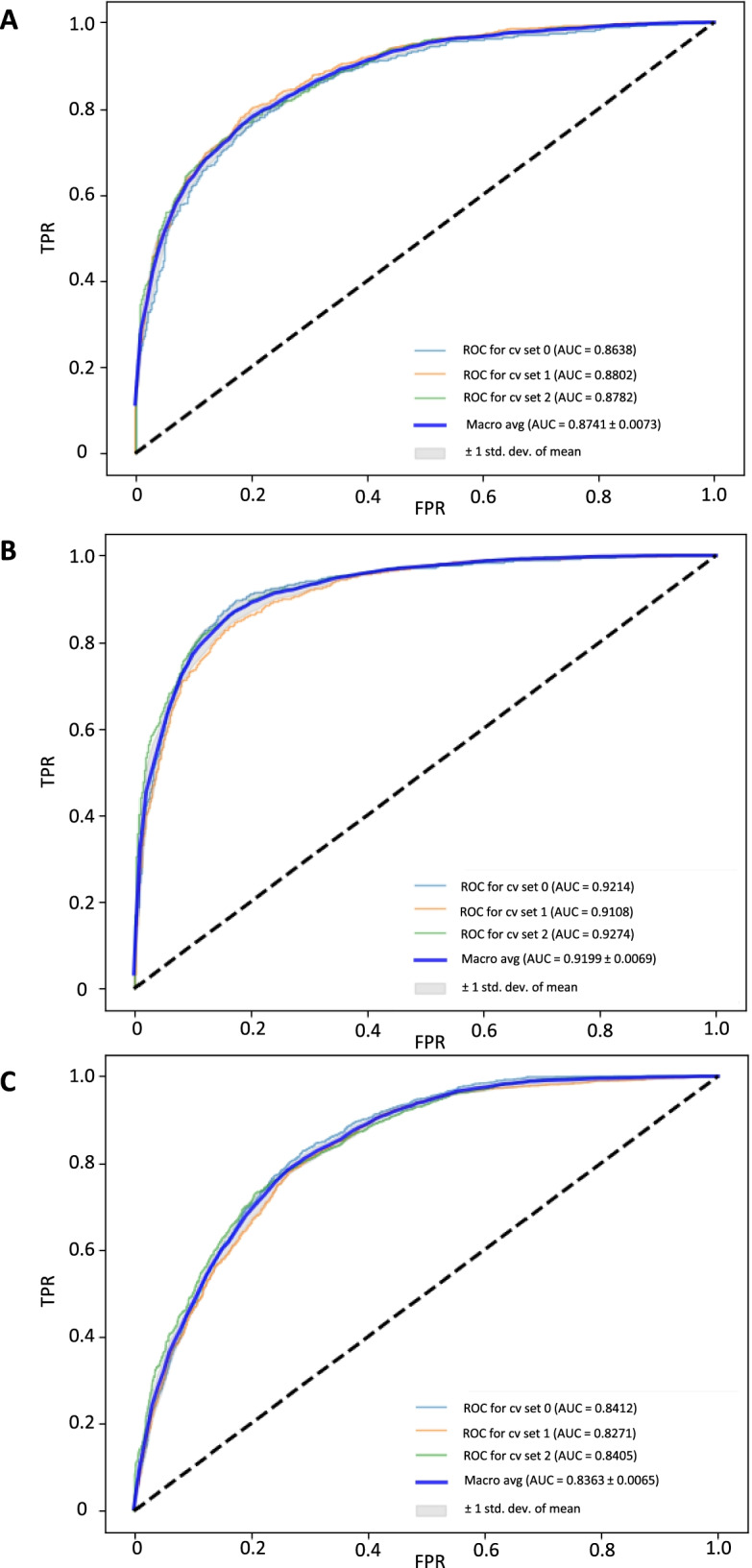
Performance of convolutional neural nets for binary cell type classifications. Since end-to-end image-level classification did not show satisfactory results in preliminary testing, we used cell-level recognition with convolutional neural nets as a proxy. Relevant cell types and features for the distinction between non-APL AML, APL and healthy bone marrow, i. e. myeloblasts, promyelocytes, and Auer rods, were labeled manually and CNNs were trained. The performance of individual CNNs for the detection of myeloblasts (**A**), promyelocytes (**B**), and Auer rods (**C**) on the respective testing sets (that were rigorously withheld from training) is displayed as area under the receiver operating curve (AUROC) using three-fold cross-validation (cv 0, 1, 2 illustrated in light blue, orange, and green). std. dev. – standard deviation of the mean; TPR – true positive rate; FPR – false positive rate

Previous efforts were conducted with a single CNN for upfront whole image classification, but showed only moderate results. The stepwise approach of distributing different classification tasks over different CNNs and integrating output information into an ENN showed substantially improved accuracy for APL prediction. Hence, we used proportions of myeloblasts, promyelocytes, and Auer rods as a proxy to achieve image-level classification. To train the CNNs, 2992 myeloblasts, 1378 promyelocytes, and 130 cells with Auer rods were labeled manually (a full list of manually labeled cells is provided in Tab. S1). For the individual binary classifications (e. g. myeloblast vs. non-myeloblast, promyelocyte vs. non-promyelocyte etc.), the CNNs’ accuracy differed (Fig. 3). The individual CNNs for the detection of myeloblasts, promyelocytes, and Auer rods showed an AUROC of 0.8741, 0.9199, and 0.8363, respectively.

**Fig. 4:**
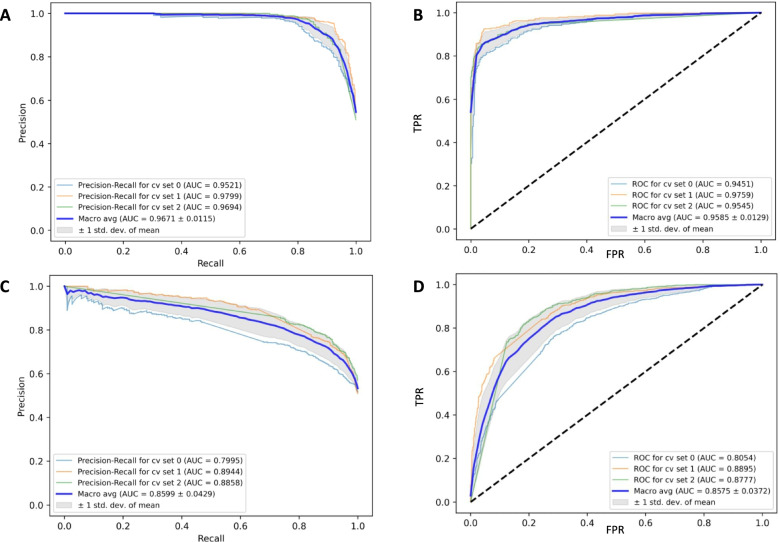
Performance of the ensemble neural net for APL image-level recognition. Performance metrics for the binary classification of APL vs. healthy bone marrow donor samples (top row) and APL vs. non-APL AML samples (bottom row) were calculated as areas under the curve for precision-recall curves (**A**, **C**) and the receiver operating characteristic (**B**, **D**) using threefold cross-validation (cv 0, 1, 2 illustrated in light blue, orange, and green) and averaging results (Macro avg, dark blue). Calculations were performed in Python. std. dev. – standard deviation of the mean; TPR – true positive rate; FPR – false positive rate

For final classifications, our ENN model achieved a mean AUC of the precision-recall-curve of 0.9671 (95%-CI: 0.9441 – 0.9901; Fig. 4A) and a mean AUC of the ROC of 0.9585 (95%-CI: 0.9327 – 0.9843; Fig. 4B) for the detection of APL samples among healthy bone marrow donor samples using threefold internal cross-validation. For the binary classification between APL and non-APL AML our ENN model reached a mean AUC of the precision-recall-curve of 0.8599 (95%-CI: 0.7741 – 0.9457; Fig. 4C) and a mean AUC of the ROC of 0.8575 (95%-CI: 0.7831 – 0.9319; Fig. 4D) with threefold internal cross-validation.

## Discussion

We here present a DL-based system for the diagnosis of APL from BMS. The resulting DL model automatically segments nucleated cells in images of BMS with high accuracy. Accurate cell segmentation is a key initial step in CNN-based evaluation of leukemic cell morphology that is considerably harder in the bone marrow as cells are often clumped in narrow spaces and artifacts are more frequent than in peripheral blood [37]. We tested APL recognition with our ENN model both against healthy bone marrow donor and non-APL AML samples and obtained an AUROC of 0.9585 and 0.8575, respectively. The time it takes from the upload of a BMS image to the model and subsequent output of a suggested diagnosis was 45.3 s. To the best of our knowledge, our model represents one of the first and the most accurate DL approach to recognize APL from bone marrow cytomorphology. The few recent studies have either focused on peripheral blood smears [38] or report a lower accuracy [39]. Given considerable early death rates especially in elderly patients [19], reliable methods for diagnosis are crucial to provide highly effective treatment. Since APL is a very rare disease, patients suspected of having APL must be referred to specialized centers as soon as possible and only experienced trained laboratory technicians and hematologists are typically able to raise the suspicion of APL, specifically in areas with low incidence rates. ML has been reported to be able to flag possible cases from peripheral blood cell counts [40] and peripheral blood smear morphology [38]. Our DL model may serve as a proof-of-concept that DL can also be implemented as a robust tool for bone marrow evaluation in rare hematologic diseases such as APL. This can be advantageous when confirmation of t(15;17) by cytogenetics or PML-RARα by FISH is still pending or when such advanced tests are not available, e. g. in peripheral centers or in developing countries without ubiquitous access to high-quality laboratory procedures and genetic testing. In such cases, our DL model may enable diagnosis of APL even in smaller centers in developing countries as long as digitization of BMS and internet access is available. For specialized centers, our system may provide a mechanism of rapidly pre-scanning samples for APL and flag them for immediate evaluation by hematopathologists.

Nevertheless, given the rarity of APL our cohort consists of only 51 cases as available training data. To account for the small sample size, we used image augmentation techniques. Indeed, to improve accuracy and further validate the model in terms of generalizability, future studies will have to include larger data sets. Especially for rare entities such as APL, international collaboration is crucial since ML models thrive on data. A larger APL data set may therefore improve the model’s accuracy and allow it to be implemented as a diagnostic screening test in clinical routine of specialized centers to pre-scan larger numbers of samples for possible cases of APL. From a technological perspective, we used cell-level detection of myeloblasts, promyelocytes and Auer rods as a proxy for subsequent BMS image-level classification by the ENNs. In preliminary experiments, we also tested direct end-to-end BMS image-level classification, although with unsatisfactory results. Conceivably, a larger data set may provide the possibility to train CNNs directly end-to-end for APL detection. Further, we tried to include a fourth CNN for the detection of faggot cells into the model, however, the scarcity of faggot cells in the sample did not allow for accurate faggot cell detection by the respective CNN. Again, a larger sample size may increase the accuracy of this individual classifier and whether a subsequent incorporation into the model could potentially boost its performance remains to be tested. While we manually selected representative BMS areas for evaluation by the DL model, future applications of the model with whole slide imaging seem warranted. Novel techniques like DL-based automated focusing on whole slide images [41] can be used to further automatize the process. However, the majority of studies of ML in hematology, including our DL approach, are developed and tested on retrospective data. Prospective validation of our DL model is planned to confirm its accuracy and transferability to daily clinical practice. We believe prospective evaluation of the model is necessary to evaluate its implications for routine diagnostics and improved accuracies given a larger training sample is needed therefore. Hence, in future iterations the model can either serve as diagnostic tool to raise awareness of suspected cases of APL for expert evaluation, provide expertise on morphologic assessment where no such expertise or access to other means of diagnostic evaluation is available and serve as a proof-of-concept that deep learning can function even with sparse data in the medical domain. We consider this to be groundwork to build upon in future iterations of the model. Furthermore, it needs to be noted that our model only considers cytomorphology and is agnostic of clinical, genetic or laboratory data. An integration of these modalities is needed to improve diagnostic accuracy and provide an even stronger decision support system for clinicians.

## Conclusion

We present a DL model for assistance in the diagnosis of APL from bone marrow cytomorphology using control cohorts of both healthy bone marrow donors and non-APL AML samples. Our ENN model achieved high values for AUROC despite the limited sample size and serves as a proof-of-concept for the viability of DL in the diagnosis of rare cancer entities from image data. Since our DL platform uses visual image data only, it may potentially be used to support diagnosis of APL in areas where molecular and cytogenetic profiling is not routinely available. Future work will therefore focus on increasing sample size, prospective validation and the implementation of an online tool for easily accessible remote use of the model’s APL prediction capabilities.

## Supplementary Information

**Additional file 1.** 

## Data Availability

The data supporting the conclusions of this article is available under https://www.kaggle.com/dataset/a49eb5eb219384adf92856e43dcfc79b9cf1eaea5ec13bd57ef304d173ebe42c All models were built in Python version 3.7.9 with Keras version 2.3.0 and TensorFlow version 2.1.2. Computations were performed using a high-performance computing system. The code supporting the conclusions of this article is available under https://github.com/SebastianRiechert/autofrcnn and https://github.com/TimSchmittmann/Fast-and-Accurate-Diagnosis-of-APL-from-BMS

## Abbreviations

AML: Acute myeloid leukemia
APL: Acute promyelocytic leukemia
BMS: Bone marrow smear(s)
CNN: Convolutional neural net
DL: Deep learning
ENN: Ensemble neural net
FISH: Fluorescence in situ hybridization
